# Environmental insults in early life and submissiveness later in life in mouse models

**DOI:** 10.3389/fnins.2015.00091

**Published:** 2015-03-31

**Authors:** Seico Benner, Toshihiro Endo, Masaki Kakeyama, Chiharu Tohyama

**Affiliations:** ^1^Laboratory of Environmental Health Sciences, Center for Disease Biology and Integrative Medicine, Graduate School of Medicine, The University of TokyoTokyo, Japan; ^2^Department of Neurochemistry, Graduate School of Medicine, The University of TokyoTokyo, Japan; ^3^Department of Neurobiology and Behavior, Nagasaki UniversityNagasaki, Japan

**Keywords:** dominance behavior, social behavior, early life environment, IntelliCage-based competition task, mouse

## Abstract

Dominant and subordinate dispositions are not only determined genetically but also nurtured by environmental stimuli during neuroendocrine development. However, the relationship between early life environment and dominance behavior remains elusive. Using the IntelliCage-based competition task for group-housed mice, we have previously described two cases in which environmental insults during the developmental period altered the outcome of dominance behavior later in life. First, mice that were repeatedly isolated from their mother and their littermates (early deprivation; ED), and second, mice perinatally exposed to an environmental pollutant, dioxin, both exhibited subordinate phenotypes, defined by decreased occupancy of limited resource sites under highly competitive circumstances. Similar alterations found in the cortex and limbic area of these two models are suggestive of the presence of neural systems shared across generalized dominance behavior.

## Introduction

Social dominance is a universal behavioral feature exhibited by social animals across species and is considered one of the few robust and reliable social behavior indices in experimental animals. Dominance behavior is exhibited primarily in competitive situations, where individuals with tendencies to dictate to others are referred to as dominant, whereas those being dictated to are referred to as subordinates (Rowell, 1974). Generally, dominant individuals gain priority of access to resources and copulation (Dewsbury, 1982; Akbaripasand et al., 2014), reflecting the ecological significance of social dominance.

Numerous intrinsic factors are thought to be involved in generating dominance behavior, such as levels of aggressiveness and anxiety (Chase et al., 2002), and dominance behavior has been used as an indicator to study affective disorders in experimental animals (Malatynska and Knapp, 2005). Although there is a substantial genetic influence determining these intrinsic characteristics (Braw et al., 2006; Malkesman et al., 2006; Babri et al., 2014), the development of social behavioral disposition is presumably nurtured by the environment as well. In particular, social environment in early life has a profound influence on the development of the social brain (Champagne and Curley, 2005) and the subsequent expression of social behaviors in adulthood (Fleming et al., 1999; Veenema, 2012; Branchi et al., 2013). Manipulations of the neonatal social environment are widely used experimental procedures in rodents and primates to investigate the developmental consequences of stress, childhood adversity, or trauma during early life. The use of such animal models has proven successful in advancing our understanding of how mother–infant and peer interactions, for instance, alter developmental trajectories. Alterations in aggressive and anxiety traits have been recognized in rats that were repeatedly isolated from their mother (maternal separation; MS) and their littermates (early deprivation; ED) during the neonatal period (Biagini et al., 1998; Marmendal et al., 2006; Rees et al., 2006) or post-weaning period (Toth et al., 2012). These dispositions arise presumably from abnormalities in the stress response system comprising the corticolimbic circuit and the hypothalamic–pituitary–adrenal (HPA) axis (Pryce et al., 2011; Birnie et al., 2013; Rincon-Cortes and Sullivan, 2014), in which its developmental programming is susceptible to stressful stimuli during critical periods in life. This observation is supported epidemiologically, with parental loss, physical abuse, sexual abuse, and neglect having been shown to be important for determining developmental outcomes, including neuroendocrine stress response (Laurent et al., 2014). Other environmental factors known to modify affective or social behavior in maturity include perinatal exposure to toxic chemicals (Disney et al., 2008; Haijima et al., 2010; Xu et al., 2012; Hamilton et al., 2014; Kiryanova and Dyck, 2014), and some of these chemical-induced behavioral abnormalities are associated with alterations in the stress response system (Glavas et al., 2007; Poimenova et al., 2010).

It is hypothesized that early life environment, particularly one that affects the neuroendocrine stress response system, shapes the neural basis of social behavior, which in turn may contribute to the hierarchical status within a group later in life. However, the contribution of the early-life environment to social dominance is largely unknown. Here we describe two mouse models that exhibit subordinate behavior in adulthood as a result of insults during development: neonatal ED manipulation (Benner et al., 2014) and perinatal exposure to an environmental pollutant, dioxin (Endo et al., 2012). We will also discuss the possible neurological foundations underlying social dominance.

## Methods for assessing dominance

Social dominance in wild animals is often determined by field observations (Gesquiere et al., 2011). Although replicating a true natural setting is a challenge in a laboratory-based experimental setup, machine-based behavioral phenotyping technologies specialized for monitoring colonies of mice have been developed (Freund et al., 2013; Ohayon et al., 2013; Weissbrod et al., 2013). They may be developed further in the near future to provide suitable tools for evaluating complex social structures such as hierarchy. Currently, however, a hierarchy is commonly assessed based on a dominant or a subordinate phenotype exhibited by one-to-one competitions, e.g., the tube test (Lindzey et al., 1961), the social interaction test (Coura et al., 2013), the urine-marking assay (Desjardins et al., 1973; Drickamer, 2001), the dominant–submissive relationship (DSR) paradigm (Feder et al., 2010), and the resident intruder test (Kaliste-Korhonen and Eskola, 2000). In other words, dominance hierarchies have been studied under the premise that dominant–subordinate relationships between pairs of individuals account for the overall hierarchical structure of a colony. Because no more than two mice can be tested at a time in the above paradigms, the efficiency of generating rankings within the tested colony is greatly compromised.

**Figure 1:**
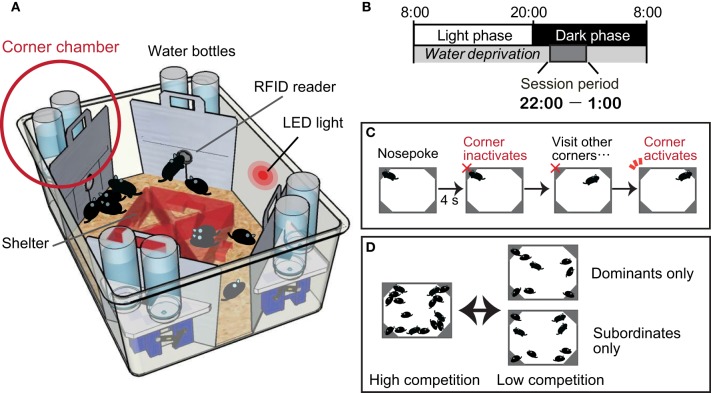
**Intelli-Cage-based competition task protocol. (A)** An IntelliCage apparatus comprising a large cage [55 × 37.5 × 20.5 cm (w × d × h)] equipped with four corner chambers [15 × 15 × 21 cm] controlled by a computer. Each of these chambers holds two water bottles and functions as a fully automated operational unit. A radiofrequency identification (RFID) device reader is located at the entrance of each chamber and enables the IntelliCage software to record the entry and exit time of each individual resident mouse, given that all resident mice have been tagged by the subcutaneous implantation of RFID microchips. An entry to each chamber is physically restricted to a single mouse at any given time. Inside the chamber, there is a motorized door in front of each water bottle nozzle. The opening and closing of the door are programmable and can be uniquely assigned for each mouse. For instance, the door can detect the nose poking behavior of a mouse, which can be used to initiate opening, and closing can be programmed by time. **(B)** Mice are deprived of water throughout the day, except during the session period between 2200 and 0100 h. Session periods are cued by an LED light on the wall of the IntelliCage, and mice are thoroughly trained to learn the cue. **(C)** Inside the chamber, a mouse uses its nose to poke either of the two doors to open it for accessing the water nozzle. The activated door is programmed to stay open for 4 s. After the door shuts, the chamber becomes inactivated for that mouse, which must go to a different corner chamber for another reward. The task protocol is thus programmed to prevent any single mouse from persistently occupying one corner chamber for an indefinite time. The occupancy of the corner chambers is measured by dwell time or visit frequency. **(D)** The experimental group composition as well as the degree of competition can be flexibly determined by adjusting the density of animals within an apparatus. Assessment of the motivation level toward reward can be achieved by dividing the dominants and subordinates into two separate cages for several days. If their visiting patterns overlap, it may be regarded as a clear indication that the motivation of the subordinate mice for drinking water is not different from that of the dominants. After the motivational level of the subordinates has been confirmed, all the mice can be combined again to confirm whether the peak number of visits in the subordinates declines once again.

We have recently established a behavioral test protocol for quantifying dominance behavior in group-housed mice (Endo et al., 2012) using a commercially available machine-based behavioral phenotyping system called an IntelliCage apparatus (Galsworthy et al., 2005) (Figure 1A). The IntelliCage-based competition task is contextually similar to the paradigm presented in the visible burrow system established for rats (Blanchard et al., 1988, 1995). In both systems, the individual animal's behaviors are assessed in a social environment, and a group of mice is subjected to a social stress resulting from competition for resources. In the visible burrow system, animals are classified as dominant or subordinate by agonistic interactions (attacks and guarding behavior) manually scored by video monitoring. In the IntelliCage-based competition task, the mice that occupy the limited resource sites at the beginning of the session are classified as dominants, while those that fail to achieve access to the resource sites are classified as subordinates.

In the competition task protocol, mice are deprived of water throughout the day, except during the 3 h session period between 2200 and 0100, to establish motivation for accessing the corner chambers for water as a reward (Figure 1B). Once a session begins, over a dozen mice compete against each other, as in a game of musical chairs, for the water in the limited access sites situated in the four corner chambers (Figure 1C). Because of the daylong water deprivation, the competition is greatest at the beginning of the session (approximately 22:00–22:05), and the occupancy of the corner chambers is monopolized by the dominant mice. During the following period (approximately 22:05–22:10), the subordinate mice can gain access to the corner chambers. After a while, the intense competition subsides. In this system, the mode of competition can be manipulated by adjusting the number of mice in a cage and the number of available corner chambers (Figure 1D).

## Competitive subordinance in group-housed mice

We have previously shown that ED mice, generated by isolating neonates from their mother and littermates for 3 h per day for the first 2 weeks after birth (Pryce and Feldon, 2003; Millstein et al., 2006), exhibit subordinate behavior in the IntelliCage-based competition task (Benner et al., 2014). We have also shown that mice perinatally exposed to a low dose of dioxin, a ubiquitous environmental pollutant, exhibit subordinate behavior in adulthood (Endo et al., 2012). In both cases, the subordinate behavior was attributable to developmental abnormality that occurred during early life, long before the time at which the behavioral tests were conducted.

The subordinate behaviors were persistently present throughout the competition task sessions for both the ED mice and the mice perinatally exposed to dioxin. A reasonable hypothesis is that the subordinate mice's motivation toward the reward is lower than that of the dominant mice, and accordingly accounts for decreased occupancy of the corner chambers. In the IntelliCage-based competition task, the level of motivation can be assessed by several means as follows: (i) evaluating the water consumption under a basal, non-competitive condition; (ii) evaluating the total dwell time and frequency of visits made within the session. If all of the mice have an equal level of motivation, an equal duration and number of total visits would be expected, although the timing of the visits may differ depending on the dominance behavior; and (iii) evaluating the subordinate mice's motivation for drinking in the absence of dominant mice (Figure 1D).

It is notable that in both mice models, the subordinate mice did not differ from the dominant mice in terms of water consumption per day and motivation for drinking water at the beginning of the water-availability period. Furthermore, the removal of the dominant mice from the cage ameliorates the subordinate mice's visiting behavior. Taken together, these observations emphasize that social environment plays an imperative role in determining the behavior of these mice, and that the early life environment can alter the vulnerability to social–emotional challenges in adulthood. The subordinate behavior may reflect a social–phobic temperament, resembling that of social anxiety disorder or autism spectrum disorder (ASD) in humans. In contrast, an abnormality in competitive dominance may be manifested in the hyperdominance of individuals, a behavior considered suggestive of conduct disorder observed in humans.

## Possible neural basis of dominance behavior

The medial prefrontal cortex (mPFC) is one of the major brain regions associated with the dominant–subordinate phenotype assessed by the IntelliCage-based competition task. This observation is consistent with previous reports on animals (Gesquiere et al., 2011; Wang et al., 2011) and humans (Zink et al., 2008; Freeman et al., 2009). In ED mice, the expression of the *Map2* gene, which is considered to be involved in dendritic remodeling associated with synaptic plasticity, is significantly reduced in mPFC, and a significant correlation is observed between the dominance level and *Map2* expression level (Benner et al., 2014). This observation is consistent with a previous report describing a significant association between dominance rank and synaptic efficiency in mPFC in mice (Wang et al., 2011). The mPFC of mice born to dams perinatally exposed to a low dose of dioxin showed reduced expression of the immediate early genes (IEGs), c-Fos and Arc, indicating reduced neuronal activity (Endo et al., 2012). The mPFC is considered to undergo experience-dependent changes. For example, social experience-related reductions in dendritic spine density and IEG expression in mPFC were found in rats exposed to ethanol during gestation (Hamilton et al., 2010). The prefrontal acetylcholine system has recently been shown to be involved in dominance behavior characterized by the social interaction test (Coura et al., 2013). In addition to the relationship of mPFC with the dominance trait, fMRI studies of humans showed that mPFC is associated with social phobia (Blair et al., 2010) and social anxiety disorders (Shang et al., 2014).

The amygdala is another brain region in which ED mice and dioxin-exposed mice share similar neurological characteristics (Endo et al., 2012; Benner et al., 2014). In both cases, c-Fos expression was elevated in the basolateral amygdala (BLA), and its expression level was inversely correlated with dominance rank in the ED study. BLA plays important regulating roles in anticipatory anxiety (Savonenko et al., 1999), social cue processing (Adolphs, 2001; Truitt et al., 2007), and stimulus–reward processing (Murray, 2007). Its function is strongly affected by early life stress both in humans (Marusak et al., 2014; Suzuki et al., 2014) and rodents (Caldji et al., 1998; Berman et al., 2014; Tzanoulinou et al., 2014). Amygdala activity habituates to repeated presentations of social stimuli in healthy subjects (Wedig et al., 2005), suggesting its role in social adaptation. However, abnormal BLA excitation has been suggested to occur in social anxiety disorder and ASD (Truitt et al., 2007; Kleinhans et al., 2009). BLA is particularly sensitive to early life stress and has a critical window (Koppensteiner et al., 2014). Children who experienced early life stress were observed to have enhanced amygdala activity (Maheu et al., 2010; Tottenham, 2012; Gee et al., 2013). Importantly, functional connectivity between the mPFC and amygdala has been recognized (Likhtik et al., 2005, 2014). The integrity of the mPFC–amygdala circuit is hypothesized to be a critical determinant of the self-regulation of socio-emotional behavior in response to one's social environment, characteristically disrupted in patients with ASD (Bachevalier and Loveland, 2006).

Social recognition and social memory are thought to contribute to the maintenance of the dominance hierarchy. Social memory, distinct from other types of memory, involves a special neural circuit relaying signals from olfactory social cues (e.g., pheromones) to the medial amygdala (MeA), which innervates the lateral septum (LS) and the bed nucleus of the stria terminalis (BNST). The neural circuit that involves the regions listed above is highly stress-responsive and regulates aggressive behavior (Ferguson et al., 2002; Nelson and Trainor, 2007). The neuropeptides vasopressin and oxytocin regulate social behavior and stress responses, and the role of oxytocin receptors in the long-term establishment of dominance hierarchies has been reported (Timmer et al., 2011).

## Stress and dominance

An association between dominance behavior and neuroendocrine stress response has been an intriguing subject in the field of social neuroscience. Experiencing dominance hierarchies can be stressful to both subordinate and dominant individuals (Blanchard et al., 1995; Gesquiere et al., 2011), and neuroendocrine characteristics associated with the stress of being subordinate have been reviewed (Blanchard et al., 1993). In general, social subordinance is associated with hypercortisolism or feedback resistance (Sapolsky et al., 1997), whereas glucocorticoid signaling is involved in agonistic behaviors, including dominance, under conditions when hierarchy has not been established. Corticosterone administration affects aggressive behavior in resident intruder conflicts (Mikics et al., 2004), but does not affect intracolony aggression in colonies that have already been established to have stable social relationships (Mikics et al., 2007). However, glucocorticoids are thought to play a critical role in the establishment of a dominance hierarchy and in the long-term maintenance of dominant–subordinate relationships. Rats exposed to stresses just before the first social encounter tend to become subordinate toward unfamiliar rats that were not exposed to the same stresses and have similar attributes, such as body weight and trait anxiety; and the dominant–subordinate relationship established between a given pair of rats persists over time (Cordero and Sandi, 2007). It is thus implied that sensitivity and reactivity toward the stress response (HPA axis function and regulation) have a major effect not only on determining the hierarchical phenotype at the time of a first social encounter but also on the long-term maintenance of an individual's dominance behavior.

Importantly, the integrity of the neuroendocrine stress response system can be modulated by external insults such as disrupted neonatal social environment and perinatal exposure to a neurotoxic chemical. Accumulating reports show that the HPA axis is programmed, at least in part, by early-life events (Matthews, 2002). In particular, early-life stress can modify the development of HPA functioning and thereby influence behavior as well as susceptibility to certain diseases in adulthood. In non-human primates, prenatal stress, experimentally induced by gestational glucocorticoid exposure, influences social play behavior and HPA axis function (Mustoe et al., 2014). Similarly, hyperaggressive traits have been observed with repeated corticosterone administration to peripubertal rats (Veenit et al., 2013).

Previous studies have shown the effects of ED on behavior in adulthood and HPA axis function (Ruedi-Bettschen et al., 2004, 2006; Marmendal et al., 2006; Rees et al., 2006, 2008). However, the developmental toxicity to the neuroendocrine stress response system of perinatal dioxin exposure has not been thoroughly assessed in mice. The HPA axis manifests acute toxicity upon dioxin exposure in primates (Shridhar et al., 2001) and rats (Balk and Piper, 1984; Bestervelt et al., 1993). For example, TCDD administration increases adrenal sensitivity to adrenocorticotropic hormone (ACTH) in adult rats (Dibartolomeis et al., 1987). In addition, pituitary gland toxicities have been shown *in vivo* (Moore et al., 1989) and *in vitro*, resulting in, for example, increases in the gene expression of the ACTH precursor proopiomelanocortin (POMC) (Bestervelt et al., 1998; Huang et al., 2000, 2002) and ACTH and corticosterone secretion (Pitt et al., 2000). Recent studies have shown that prenatal dioxin exposure reduces the expression of pituitary hormones (Takeda et al., 2014) and decreases the circulating level of corticosterone in pregnant dams and their fetuses. This response causes *in utero* growth retardation that can be rescued by supplying corticosterone to dioxin-exposed dams (Hattori et al., 2014). These findings suggest that the HPA axis is disrupted in the perinatal dioxin exposure model.

## Conclusions

We have described two cases in which early-life environmental manipulations have induced alterations in dominance behavior. These studies extend previous observations that social behavior can be shaped by environment, and show that competitive dominance is a robust, reliable, and also highly sensitive trait allowing the evaluation of the effects of developmental insults on neuroendocrinological systems in mice. Dominance is presumably more complex than one-to-one competition and is highly dependent on the social environment. The IntelliCage-based competition task permits the determination of the individual mouse's level of dominance in a group, given that the task is presented simultaneously to over a dozen mice in a single apparatus. Thus, it is considered that the dominance in this test represents not merely competitive but social dominance. In addition, an evaluation of the correlation between the level of dominance and the gene expression patterns in the ED model cannot be achieved by other standardized behavioral assays used to investigate the social status in rodents.

## Author contributions

SB, TE, MK, and CT wrote the paper.

### Conflict of interest statement

The authors declare that the research was conducted in the absence of any commercial or financial relationships that could be construed as a potential conflict of interest.
